# ENSO and Southeast Asian biomass burning modulate subtropical trans-Pacific ozone transport

**DOI:** 10.1093/nsr/nwaa132

**Published:** 2020-06-13

**Authors:** Lian Xue, Aijun Ding, Owen Cooper, Xin Huang, Wuke Wang, Derong Zhou, Zhaohua Wu, Audra McClure-Begley, Irina Petropavlovskikh, Meinrat O Andreae, Congbin Fu

**Affiliations:** Joint International Research Laboratory of Atmospheric and Earth System Sciences, School of Atmospheric Sciences, Nanjing University, Nanjing 210023, China; Jiangsu Provincial Collaborative Innovation Center for Climate Change, Nanjing 210023, China; Joint International Research Laboratory of Atmospheric and Earth System Sciences, School of Atmospheric Sciences, Nanjing University, Nanjing 210023, China; Jiangsu Provincial Collaborative Innovation Center for Climate Change, Nanjing 210023, China; Cooperative Institute for Research in Environmental Sciences, University of Colorado, Boulder, CO 80305, USA; Chemical Sciences Division, NOAA Earth System Research Laboratory, Boulder, CO 80305, USA; Joint International Research Laboratory of Atmospheric and Earth System Sciences, School of Atmospheric Sciences, Nanjing University, Nanjing 210023, China; Jiangsu Provincial Collaborative Innovation Center for Climate Change, Nanjing 210023, China; Joint International Research Laboratory of Atmospheric and Earth System Sciences, School of Atmospheric Sciences, Nanjing University, Nanjing 210023, China; Jiangsu Provincial Collaborative Innovation Center for Climate Change, Nanjing 210023, China; Joint International Research Laboratory of Atmospheric and Earth System Sciences, School of Atmospheric Sciences, Nanjing University, Nanjing 210023, China; Jiangsu Provincial Collaborative Innovation Center for Climate Change, Nanjing 210023, China; Joint International Research Laboratory of Atmospheric and Earth System Sciences, School of Atmospheric Sciences, Nanjing University, Nanjing 210023, China; Department of Earth, Ocean and Atmospheric Sciences, Florida State University, Tallahassee, FL 32306, USA; Cooperative Institute for Research in Environmental Sciences, University of Colorado, Boulder, CO 80305, USA; Global Monitoring Division, NOAA Earth System Research Laboratory, Boulder, CO 80305, USA; Cooperative Institute for Research in Environmental Sciences, University of Colorado, Boulder, CO 80305, USA; Global Monitoring Division, NOAA Earth System Research Laboratory, Boulder, CO 80305, USA; Max Planck Institute for Chemistry, Mainz 55128, Germany; Scripps Institution of Oceanography, University of California, San Diego, La Jolla, CA 92093, USA; Joint International Research Laboratory of Atmospheric and Earth System Sciences, School of Atmospheric Sciences, Nanjing University, Nanjing 210023, China; Jiangsu Provincial Collaborative Innovation Center for Climate Change, Nanjing 210023, China

**Keywords:** El Niño-Southern Oscillation (ENSO), climate, biomass burning, tropospheric ozone, long-range transport, Southeast Asia

## Abstract

Trans-Pacific transport of enhanced ozone plumes has been mainly attributed to fossil fuel combustion in Asia in spring, but less attention has been paid to vegetation fires in Asia. Here we show that the El Niño-Southern Oscillation (ENSO)-modulated fires in Southeast Asia, rather than Asian fossil fuel plumes, dominate the interannual variability of springtime trans-Pacific transport of ozone across the entire North Pacific Ocean. During El Niño springs, the intensified fires from both the Indochinese Peninsula and Indonesia, together with large-scale circulation anomalies, result in enhanced ozone plumes that stretch over 15 000 km in both the lower-middle and upper troposphere. This enhancement is also observed in the *in situ* measurements of ozone concentration, with an almost 10% increase at Mauna Loa Observatory, Hawaii, a unique site to monitor the long-distance transport over the North Pacific. This study reports an unexpectedly strong influence of vegetation fires, linked with climate variability, on global tropospheric chemistry and proves once more how complex the interactions in the climate system are.

## INTRODUCTION

Ozone (O_3_), together with the hydroxyl radical (OH), dominates the atmospheric oxidizing capacity and cycling of reactive trace gases in the troposphere, and thereby plays a key role in atmospheric chemistry and climate change [1–6]. As a result of its lifetime of a few weeks in the free troposphere, O_3_ can be transported on regional to intercontinental scales [2,7–11]. Previous studies of long-range transport of O_3_ and its precursors from Asia have mainly focused on anthropogenic fossil fuel (FF) combustion sources [7,10–14]. Intercontinental transport from Asia to North America at mid-latitudes is particularly strong in spring because of frequent cold fronts related to a favorable position of the jet stream [5,8,9,15,16]. Here we instead focus on the subtropics and show that the springtime biomass burning (BB) emissions in Southeast Asia, including the Indochinese Peninsula and Indonesia, dominate the O_3_ interannual variability across the subtropical North Pacific Ocean under the influence of the El Niño-Southern Oscillation (ENSO).

## RESULTS

### Interannual variability of O_3_ over the subtropical North Pacific Ocean

**Figure 1.**
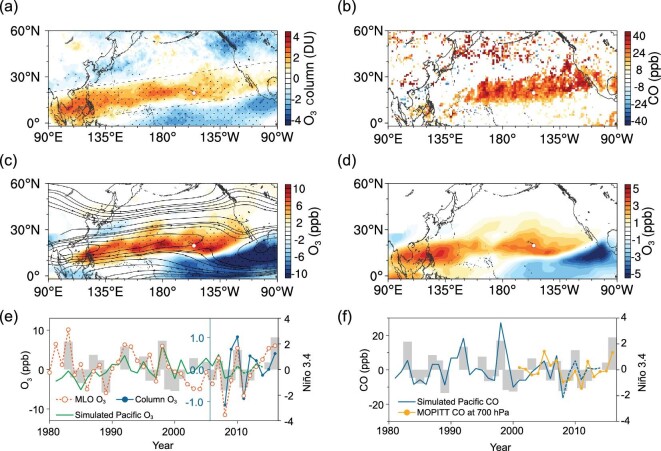
ENSO-induced O_3_ and CO enhancements over the subtropical North Pacific Ocean in spring. (a) Difference of IASI retrieved 0–6 km O_3_ column in El Niño and La Niña springs. (b) Difference of MOPITT retrieved 700 hPa CO between El Niño and La Niña springs. Only statistically significant data (*P* < 0.1) are shown. (c) Simulated climatological streamlines and ENSO-induced O_3_ difference at 680 hPa (the results were deduced based on ElNino_BASE and LaNina_BASE runs given in Supplementary Table 1). (d) The first EOF mode of spring O_3_ at 680 hPa in 1981–2008 (from the FSDSMAM-hist Experiment). (e) Simulated O_3_ over the subtropical Pacific (averaged for the dashed box in (a)), observed O_3_ at Mauna Loa Observatory (MLO), satellite retrieved O_3_ column in spring, and Niño 3.4 index averaged in the preceding December–February (DJF) (gray bars). (f) Simulated and MOPITT observed CO over the subtropical Pacific in spring and Niño 3.4 index averaged in the preceding DJF. The dotted areas in (a) and (c) indicate statistical significance with 90% confidence.

Figure 1 shows how the springtime distribution of O_3_ and its precursors across the subtropical North Pacific Ocean are linked to ENSO. The IASI satellite retrievals show that during El Niño, a positive anomaly of tropospheric O_3_ (below 6 km) extends in spring from the South China Sea northeastward to southwestern North America and the Gulf of Mexico (Fig. 1a). This belt of O_3_ anomalies, with an average value of 1.7 DU, has a width of about 1500 km and a length of over 15 000 km, far longer than the mid-latitude intercontinental transport pathways from East Asia to North America and from eastern North America to Europe [17,18]. As a precursor of O_3_ and a tracer for combustion sources [19], carbon monoxide (CO) from MOPITT satellite retrievals also shows an anomalous distribution during El Niño springs (Fig. 1b).

Simulations using the Community Earth System Model (CESM) CAM-Chem [20] in specified dynamics mode based on the MERRA reanalysis for the period 1981–2015 capture well the positive O_3_ anomaly (Fig. 1c). By applying Empirical Orthogonal Function (EOF) analysis, we find that the first principal component (PC1) of the simulated springtime O_3_ given in Fig. 1d is consistent with the O_3_ distributions in Fig. 1a and c, and can explain 25% of the O_3_ variability in the subtropical North Pacific (Supplementary Fig. 1). The time series of O_3_ and CO from satellite retrievals and model simulations in the marked region across the northern subtropical Pacific (defined in Fig. 1a), as well as the EOF results, all consistently show in-phase fluctuations with the pre-spring (December–February) ENSO index Niño 3.4 [21] (Fig. 1e and f, Supplementary Fig. 1).

**Figure 2.**
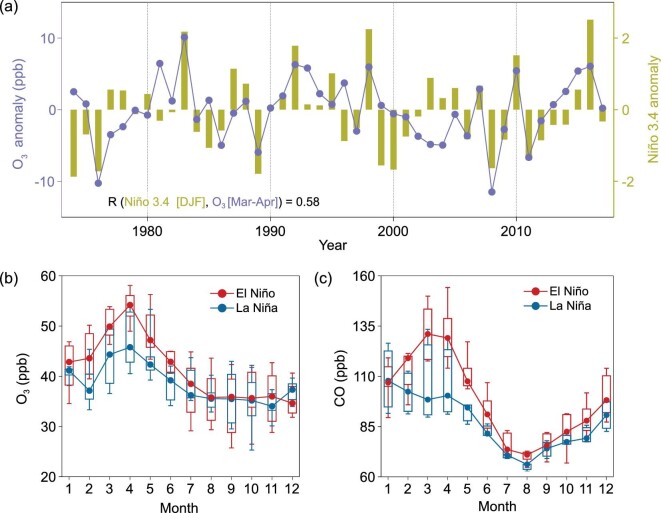
Impact of ENSO on O_3_ and CO at Mauna Loa Observatory. (a) Springtime (March–April) O_3_ anomaly at MLO and Niño 3.4 anomaly of the preceding winter (December–February). Local time 0:00–8:00 data are averaged to represent the ozone level of free tropospheric air. Seasonal variations of MLO O_3_ (b) and CO (c) in El Niño and La Niña years (defined by the 10 highest and 10 lowest years in the Niño 3.4 index, respectively). Whiskers in (b) and (c) show the 25–75% data range and bars give the 10th and 90th percentiles.

Figure 1a–d shows that the belts of O_3_ and CO anomalies intersect Mauna Loa Observatory (MLO), Hawaii, a well-known Global Atmosphere Watch (GAW) station with long continuous records of ambient baseline carbon dioxide and O_3_ [12,22]. The boreal spring (March and April) O_3_ concentrations at MLO correlate with the pre-spring Niño 3.4 index (Fig. 1e). A comparison of the mean seasonal cycles of O_3_ and CO between selected El Niño and La Niña years clearly shows that the ENSO-induced anomalies in O_3_ and CO occur mainly in spring, with particularly large differences in March and April of about 7 ppb and 30 ppb, respectively (Fig. 2).

### Sea surface temperature (SST) anomalies shift atmospheric circulations and modulate spring O_3_

**Figure 3.**
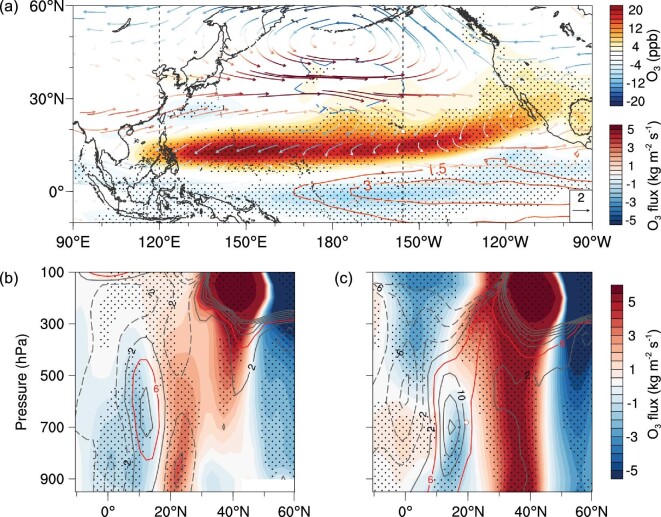
ENSO-induced anomalies in O_3_ and its flux simulated by SST-driven CESM. (a) Simulated springtime O_3_ difference at 680 hPa between ElNino_SST and LaNina_SST runs. Isolines show prescribed SST difference in the preceding DJF. Vectors are horizontal wind anomalies, colored by O_3_ flux difference at 680 hPa. Latitude cross-section of the difference in O_3_ (contours) and its flux (in isolines by 1 kg m^−2^ s^−1^) between ElNino_SST and LaNina_SST runs at 120°E (b) and 155°W (c), indicated by the dashed line in (a). The dotted areas indicate statistical significance with 90% confidence.

ENSO is known to be one of the main processes that regulates the interannual variability of the atmospheric circulation [23–26], and has been found to exert a strong impact on tropospheric ozone and other chemical constituents in tropical regions and in the Northern Hemisphere mid-latitudes [27–29]. To further isolate the impact of ENSO on the O_3_ enhancement, we conducted two sensitivity runs of CAM-Chem forced with composite typical ENSO SST with climatological emissions [21,30] (Supplementary Table 1). The O_3_ differences between the two runs (Fig. 3a) are consistent with the satellite measurements and the simulations nudged with the MERRA data (Fig. 1a and c), indicating that the ENSO SST anomaly is the main driver of the shifting circulation patterns that influence the inter-annual variability of lower tropospheric O_3_ across the subtropical North Pacific Ocean. The ENSO-induced wind-vector anomalies clearly indicate enhanced continental outflow from the Asian subtropics and mid-latitudes (20°N–40°N) to the central Pacific (Fig. 3). Figure 3a also suggests an ENSO-induced enhancement of anti-cyclonic circulation in the subtropical western Pacific, which may cause more descending motion and stronger solar radiation and hence enhanced chemical production of O_3_, despite a relatively weak CO anomaly in the western Pacific (Fig. 3a, Supplementary Fig. 2a).

The vertical cross-section of the differences in O_3_ fluxes along 120°E between the two runs clearly shows two isolated centers of outflow anomalies from Asia (Fig. 3b and Supplementary Fig. 2b). In the lower troposphere, the dominant O_3_ and CO fluxes in the western Pacific exist around 26°N (Fig. 3b), corresponding to the anomaly of northeastward transport in the coastal region of southern China (Fig. 3a, Supplementary Fig. 2a). Further south, near 10°N–15°N, the O_3_ enhancement in El Niño spring is associated with stronger westward transport (i.e. negative flux) of the recirculated aged plume. In the central-eastern Pacific (i.e. along 155°W), the ENSO induced tropospheric O_3_ enhancement has a maximum value around 700 hPa between 10°N and 25°N, associated with stronger westward transport of aged continental flows recirculated from Asia (Fig. 3a and c). Interestingly, MLO is located at the key location where the flow bifurcates to the west and the east horizontally, and where the enhancement reaches its maximum vertically, making it a key site for monitoring the ENSO-induced long-range transport of Asian pollutants.

### Stronger biomass burning in ENSO years dominates spring O_3_ enhancement

Previous studies [7,12,31,32] revealed transport pathways of Asian FF pollutants across the North Pacific Ocean. In our study, the enhanced transport flux of O_3_ and CO plumes from tropical and subtropical Asia are consistent with the previously reported pathways of Southeast Asia BB plumes [8,9], which are particularly strong in spring [8]. In addition, the BB emission intensity in this region is also linked with ENSO because of reduced precipitation during El Niño years [27,33]. As shown in Supplementary Fig. 3 and Supplementary Table 2, the BB emission intensity can be up to 57% higher in El Niño years. Lagrangian backward dispersion modelling indicates that during the El Niño springs, air masses at MLO experience a greater residence time above South and Southeast Asia (Supplementary Fig. 4). Accordingly, measurements at MLO in spring show a good correlation between O_3_ and CH_3_Cl, an effective BB tracer, and the Lagrangian modelling results clearly establish a link to the main BB source regions in tropical and subtropical Asia (Supplementary Fig. 5). The springtime maxima of O_3_ and CO at MLO (Fig. 2b and c) also indicate a ΔO_3_/ΔCO ratio of about 0.3, which is quite typical of aged BB plumes [34,35].

**Figure 4.**
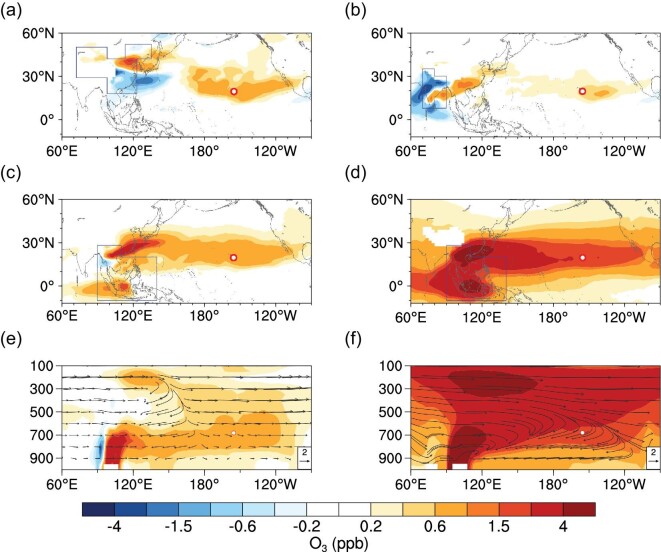
Contributions of ENSO-induced O_3_ anomalies from main Asian emission sources in spring. Averaged O_3_ response to anthropogenic emissions over China (a) and India (b) in El Niño and La Niña springs (March and April) at 680 hPa. (c) Averaged O_3_ response to climatological BB emissions over SE Asia under ENSO circulations in El Niño and La Niña springs at 680 hPa. (d) Averaged O_3_ response to ENSO-induced emission intensity variability over SE in El Niño and La Niña springs at 680 hPa. (e, f) Cross-sections of O_3_ anomalies over 15°N–25°N, corresponding to (c) and (d). The vectors are climatological springtime winds in (f) and wind anomalies between El Niño and La Niña springs in (e). Note that the vertical components of wind are scaled 1000 times for better illustration. Source regions in (a–d) are denoted by blue polygons. Red circles mark the location of MLO.

**Figure 5.**
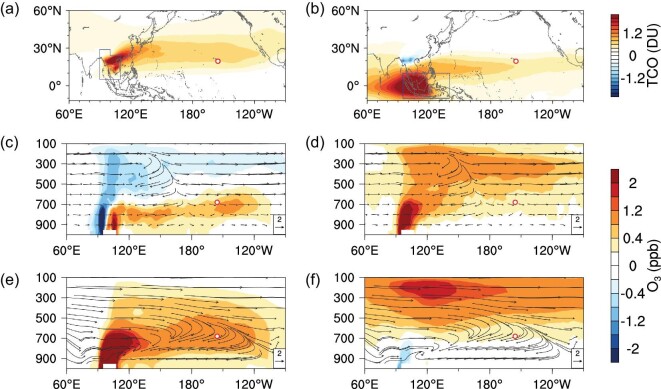
Comparison of ENSO-induced O_3_ anomalies from biomass burning in the Indochinese Peninsula and Indonesia in spring. Averaged response of the total column O_3_ (TCO) below 200 hPa to averaged BB emissions from the Indochinese Peninsula (a) and Indonesia (b) in El Niño and La Niña springs. (c–e) Cross-section of O_3_ anomalies averaged over 15°N–25°N caused by ENSO-induced circulation differences (with climatologically averaged BB emission intensity). (d–f) Cross-section of averaged O_3_ anomalies by ENSO-related BB emission differences over the Indochinese Peninsula (d) and Indonesia (f). Source regions are denoted by blue polygons. Red circles mark the location of MLO. Source contributions were calculated as listed in Supplementary Table 1.

**Figure 6.**
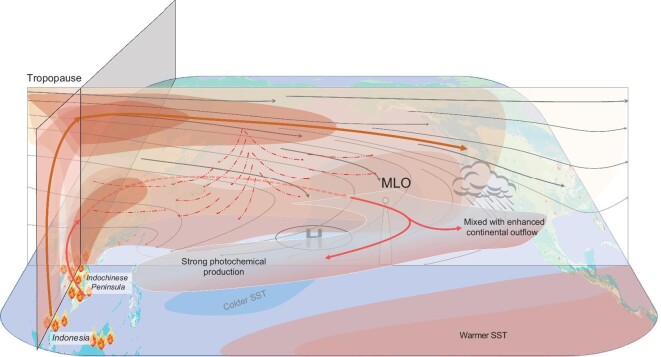
Conceptual schematic of the trans-Pacific O_3_ enhancement induced by ENSO and ENSO-mediated vegetation fires in Southeast Asia in spring. The vertical cross-section shows the O_3_ plumes and wind flows (gray lines for climatological average and red dashed ones for the anomaly in El Niño spring). Red and brown bold lines show the main transport pathways of biomass burning plumes from the Indochinese Peninsula, and Indonesia, respectively. The red dashed lines present the ENSO-induced wind anomalies along the cross-section. Blue and pink areas over the Pacific indicate the SST anomaly between the El Niño and La Niña years.

We further conducted a series of CAM-Chem simulations (Supplementary Table 1) to quantify the relative contributions from major source regions in Asia. The FF emissions in China mainly cause a positive O_3_ anomaly in the eastern Pacific (Fig. 4a), consistent with the wind anomaly shown in Fig. 3a, while those from India cause a weak anomaly in the eastern Pacific, but a stronger anomaly over southern China and southern India (Fig. 4b). However, for BB emissions in Southeast Asia, both the ENSO-induced anomalies in circulation and enhanced BB emissions make stronger contributions to the O_3_ and CO anomalies stretching from subtropical Asia to North America (Fig. 4c and d, Supplementary Figs 6 and 7). The ENSO-induced circulation anomaly causes enhancements of O_3_ and CO in the lower-middle troposphere (between 600 and 800 hPa), with a bifurcated wind flow anomaly in the central Pacific (Fig. 4e, Supplementary Fig. 6e). However, the difference in BB emissions produces a stronger impact over the subtropical Pacific, with two isolated strong vertical anomalies in the lower troposphere and the upper troposphere, for both O_3_ and CO (Fig. 4f and Supplementary Fig. 6f). The enhanced BB emissions from the Indochinese Peninsula and Indonesia in El Niño springs control the O_3_ anomalies in lower-middle troposphere and the upper troposphere, respectively, over the entire subtropical North Pacific Ocean (Fig. 5). These results are consistent with the vertical O_3_ anomalies revealed by ozonesonde profiles from Hilo, Hawaii (close to MLO) (Supplementary Fig. 8), located between the two belts of tropospheric O_3_ column enhancements from Indonesia and the central-eastern Pacific, respectively (Fig. 5a and b). The detailed transport mechanism and key processes of the BB smoke transport from the Indochinese Peninsula and Indonesia that influence the O_3_ enhancement over the subtropical Pacific are illustrated in Fig. 6.

## CONCLUSION

Our results have expanded understanding of the intercontinental-scale transport of Asian pollutants to North America [7,12,31,32] and of the roles of interannual variability in emissions and meteorology on tropospheric O_3_ [14,27,36]. We find that ENSO plays a greater role than previously thought as a driver of the interannual variability of both lower-tropospheric and upper-tropospheric O_3_ over the subtropical North Pacific Ocean and its subsequent transport to North America. In particular, our work shows that the year-to-year difference in BB emissions modulated by ENSO makes a stronger contribution than the anomalies because of meteorological variability alone. It proves once more how complex the interactions in the climate system are. This study highlights the importance of continuous measurements in the remote North Pacific Ocean for characterizing the impacts from both natural climate variability and human activity, and also suggests that better ENSO forecasting could improve modelling of continental-scale long-range transport of air pollutants.

## MATERIALS AND METHODS

### Observations, data and ENSO events definition

Ambient ozone mixing ratios have been measured at MLO since 1973 [37] and the MLO CO record covers 1989 to the present [38]. To exclude possible influences from daytime upslope winds [22], our analysis is restricted to nighttime (0:00–8:00 local time) observations.

We use the monthly 0–6 km column O_3_ dataset retrieved by Infrared Atmospheric Sounding Interferometer (IASI) [39] from 2008 to 2017 to compute the springtime column O_3_ composites in different ENSO phases. Daytime CO profiles from the Measurements of Pollution in the Troposphere (MOPITT) [8,40] are used to compute the CO composite maps under El Niño and La Niña conditions during 2000–2014.

We use the Empirical Orthogonal Function (EOF) analysis to objectively identify the dominant modes of variability of springtime (Mar–Apr) tropospheric O_3_. This technique is applied on model-simulated O_3_ at 680 hPa from 1981 to 2008 over 20°S–60°N, 60°E–90°W.

Niño 3.4 index is used to represent the ENSO evolution. Ten El Niño events and 10 La Niña events are identified in this study. The definition of ENSO events is described in the Supplementary data.

### Simulations with CESM model

We use the Community Atmosphere Model Version 5 with Chemistry (CAM5-Chem) [41] of the National Center for Atmospheric Research (NCAR) Community Earth System Model (CESM) version 1.2.2 to examine the response of the atmospheric circulation and chemical processes to ENSO events. Two sensitivity experiments, with SSTs prescribed to El Niño and La Niña conditions, and a series of simulations in specified dynamics mode, with meteorological fields nudged to the Modern Era-Retrospective Analysis for Research and Applications (MERRA) reanalysis dataset [42], have been conducted in this study. Detailed information of model description, experiments configurations and emission scenarios of these experiments are described in the Supplementary data.

### Lagrangian particle dispersion modeling

Lagrangian particle dispersion modeling is applied to demonstrate the transport pathways and track the potential sources of air masses recorded at MLO based on the Hybrid Single Particle Lagrangian Integrated Trajectory (HYSPLIT) model [43]. Three-thousand particles were released every hour at 3400 m above sea level over MLO, which are then tracked backward in time for 20 days. The footprint of the retroplume, which is represented by the residence time of particles below 100 m [44], is used to identify potential emissions source regions and to gauge the relative impact of the emissions at MLO.

### Data and materials availability

The observations, met data and modeling codes are freely available. Measurements from MLO are available from the NOAA Global Monitoring Division (GMD). Methyl chloride data from MLO were provided by S. Montzka, NOAA.

## Supplementary Material

nwaa132_Supplemental_File
