# Supplementary material for: ENSO and Southeast Asian biomass burning modulate subtropical trans-Pacific ozone transport
Source: Natl Sci Rev. 2020 Jun 13;8(6):nwaa132. doi: 10.1093/nsr/nwaa132 (PMC8288171; doi:10.1093/nsr/nwaa132)
Supplement: nwaa132_Supplemental_File [file nwaa132_supplemental_file.docx]

Supplementary Materials for

**ENSO and Southeast Asian biomass burning modulate subtropical trans-Pacific ozone transport**

Lian Xue^1,2^, Aijun Ding^1,2*^, Owen Cooper^3,4^, Xin Huang^1,2^, Wuke Wang^1,2^, Derong Zhou^1,2^, Zhaohua Wu^5,1^, Audra McClure-Begley^3,6^, Irina Petropavlovskikh^3,6^, Meinrat O. Andreae^7,8^, and Congbin Fu^1,2^

^1^Joint International Research Laboratory of Atmospheric and Earth System Sciences, School of Atmospheric Sciences, Nanjing University, Nanjing, 210023, China.

^2^Jiangsu Provincial Collaborative Innovation Center for Climate Change, Nanjing, 210023, China.

^3^Cooperative Institute for Research in Environmental Sciences, University of Colorado, Boulder, CO80305, USA.

^4^NOAA Earth System Research Laboratory, Chemical Sciences Division, Boulder, Colorado, CO80305, USA.

^5^Department of Earth, Ocean and Atmospheric Sciences, Florida State University, Tallahassee, FL32306, USA.

^6^NOAA Earth System Research Laboratory, Global Monitoring Division, Boulder, Colorado, CO80305, USA.

^7^Max Planck Institute for Chemistry, 55128 Mainz, Germany.

^8^Scripps Institution of Oceanography, University of California San Diego, CA92093, USA

*Correspondence to: dingaj@nju.edu.cn

This file contains:

1. Supplementary Text.
2. Supplementary Figures 1-9.
3. Supplementary Tables 1-2.

**Supplementary Text**

**ENSO events definition**

We use the monthly SST anomaly over 5 °S - 5 ^o^N, 120-170 ^o^W, known as the Niño 3.4 Index developed by the NOAA Climate Prediction Center, to represent the ENSO evolution. Based on the definition of a 5 consecutive 3-month running mean of SST anomalies above (below) the threshold of +0.5 °C (-0.5 °C), 10 El Niño events are identified in this study (1982/83, 1986/87, 1987/88, 1991/92, 1994/95, 1997/98, 2002/03, 2004/05, 2006/07, 2009/10) and 10 La Niña events (1983/84, 1984/85, 1988/89, 1995/96, 1998/99, 1999/2000, 2005/06, 2007/08, 2010/11, 2011/12). To examine the impacts of ENSO events with strong intensity and typical evolution, we choose 5 typical El Niño (1982/83, 1991/92, 1997/98, 2002/03, 2009/10) and La Niña events (1988/89, 1995/96, 1999/2000, 2007/08, 2010/11) for detailed comparisons. Also, we defined the springtime periods following Niño 3.4 peak winters as El Niño springs due to the persistence of the SST patterns and their continuing impacts.

**CESM Model and simulation description**

NCAR CESM 1.2.2 with CAM5-Chem model is used in this study to examine the response of the atmospheric circulation and chemical processes to ENSO events. The CAM5-Chem model includes a comprehensive tropospheric chemical mechanism, mostly adopted from the Model for Ozone and Related chemical Tracers (MOZART) version 4 mechanism for the troposphere. It also includes stratospheric chemistry. The horizontal resolution is 1.9° by 2.5°, and the vertical resolution varies with different configurations, which will be clarified below. The anthropogenic and BB emission inventories are from Monitoring Atmospheric Composition and Climate/CityZen (MACCity). The ENSO-induced difference in BB emissions is based on statistics of the MACCity inventory in years with ENSO events.

Two sensitivity experiments have been conducted with SSTs prescribed to El Niño (ElNino_SST run) and La Niña (LaNina_SST run) conditions, respectively. The El Niño/La Niña SSTs are obtained by an average of SSTs during the 5 strong El Niño/La Niña events described above. To simulate a full-term evolution of SST of an El Niño/La Niña event, two-years of SSTs are used for each event. Each experiment is initialized with 5 years of climatological SSTs for a spin-up, and then run for 2 years with El Niño or La Niña SSTs. To reduce model uncertainty, each experiment comprises 5 ensemble members, which differ from each other only in initial conditions. The vertical resolution is 30 hybrid levels up to 3 hPa (around 40 km). The BB emission inventories are fixed to climatology cycles averaged in 1980-2008. The anthropogenic emissions are averaged for 2004-2008.

We also conducted a series of simulations in specified dynamics mode, with winds, air temperature, surface pressure, and heat flux nudged to the Modern Era-Retrospective Analysis for Research and Applications (MERRA) reanalysis dataset. The internally computed meteorological fields are nudged by 10% towards MERRA reanalysis every time step (30 min). The vertical resolution is 56 levels, from the surface to about 40 km, consistent with the MERRA dataset. Two groups of simulations are employed using different meteorological conditions. The first group uses meteorological fields from the five strong El Niño years as described above. Each run in this group is an average of 5 simulations driven by meteorological conditions from 5 individual events. Similarly, the second group is based on meteorological fields from 5 La Niña events.

We then conducted a series of simulations with different anthropogenic and BB emission scenarios to identify the relative contribution of different emissions from different regions. The first run sets anthropogenic and BB emissions to 2006 conditions (a neutral case) and is used as a control run (ElNino_BASE). The ElNino_ENSEBB, ElNino_LNSEBB, Elnino_SEclmBB and Elnino_noSEBB runs use the same settings as the ElNino_BASE run, except that the BB emissions over SE Asia are set to El Niño conditions, climatology and zero, respectively. Similar parallel runs have been set for La Niña years (Supplementary Table 1). The differences between these runs are used to estimate contributions of BB over SE Asia to O_3_ variations under El Niño dynamics.

Another three runs are used to identify the individual contributions of two key regions with intense anthropogenic emissions (China and India). The ElNino_noChina and ElNino_noIndia runs use the same settings as the ElNino_BASE run, except that the anthropogenic emissions from China and India are excluded, respectively. A comparison of these three runs to the ElNino_BASE run demonstrates the relative contribution of anthropogenic emissions from these three regions under El Niño conditions.

All runs described above in the El Niño group have parallel experiments in the other group conducted under La Niña dynamical conditions with climatological FF emissions and La Niña BB emissions. A comparison of the parallel runs in the two groups reveals the modification of El Niño/La Niña dynamics to regional anthropogenic or BB contributions.

A hindcast simulation (FSDSMAM-hist) is conducted over 1981-2008, which used configurations of CAM4-chem and meteorological fields nudged towards fields from the MERRA reanalysis product. Yearly anthropogenic and BB emissions are also from the MACCity emission dataset.

Model configurations and emission scenarios of these experiments are detailed listed in Supplementary Table 1.


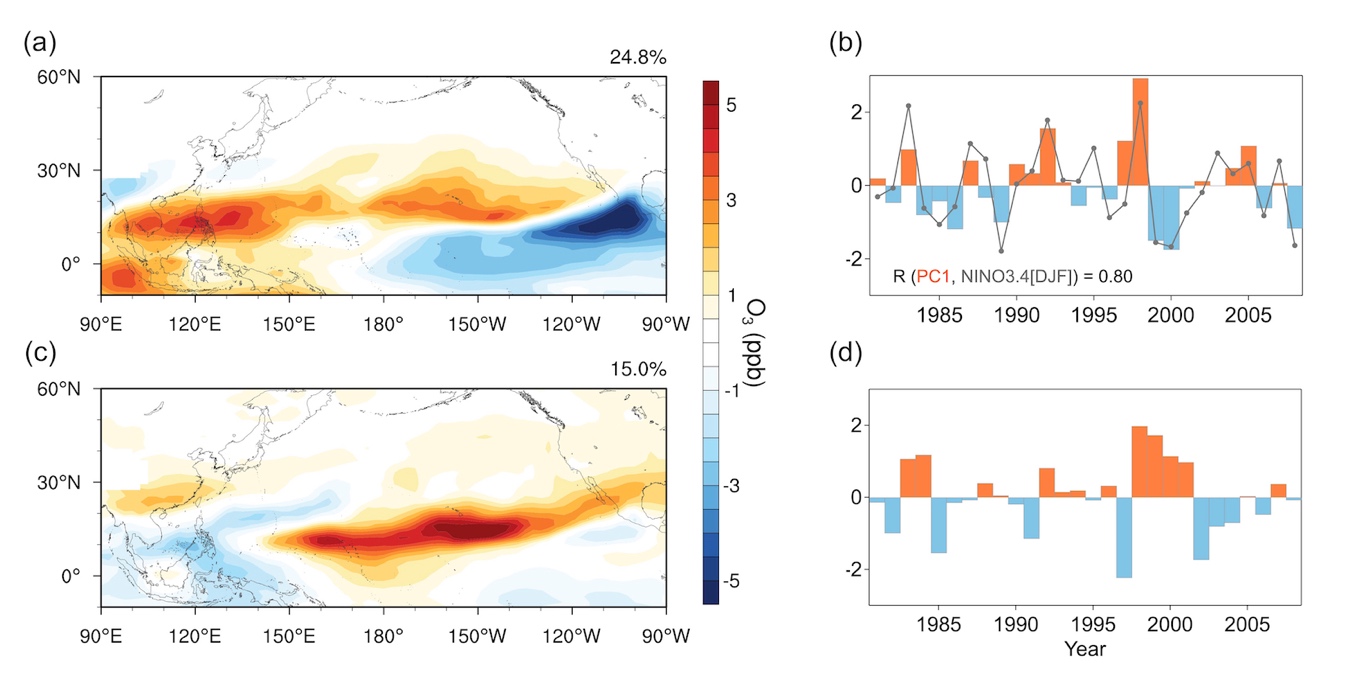


**Supplementary Figure 1.** Interannual to decadal signal in long-term simulated ozone by EOF decomposition. (a), (c) The first two EOF modes and (b), (d) the corresponding principal components (PCs) decomposed from FSDSMAM-hist simulations. The percentage variance (units: %) explained by each EOF is shown above each plot of the left panels. The line with grey markers in (b) is the Niño 3.4 index of the preceding winter (Dec-Jan-Feb). The correlation coefficient R between PC1 and the NINO3.4 index of the preceding DJF is shown in (b).


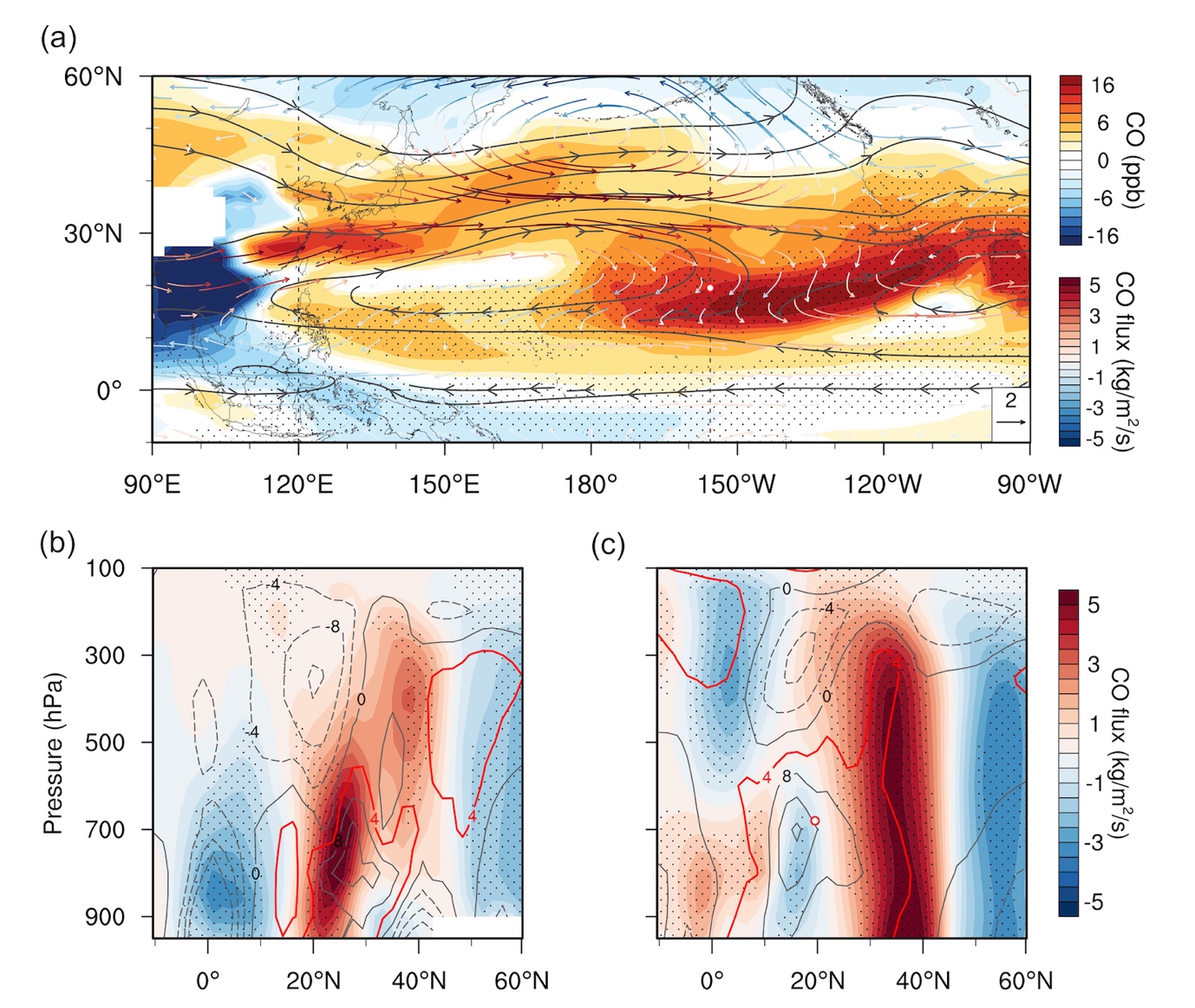


**Supplementary Figure 2.** ENSO-induced anomalies in CO concentration and flux simulated by SST-driven CESM. (a) Simulated springtime CO difference at 680 hPa between ElNino-SST and LaNina-SST simulations. Isolines show prescribed SST difference in the preceding DJF. Vectors are horizontal winds anomalies, colored by CO flux difference at 680 hPa. (b)-(c) Latitude cross section of difference in CO (contours) and CO flux (in isolines by 1 kg/m^2^/s) between ElNino-SST and LaNina-SST simulations at 120 °E (b) and 155 °W (c), indicated by the dashed line in (a). The dotted areas indicate statistical significance with 90% confidence.


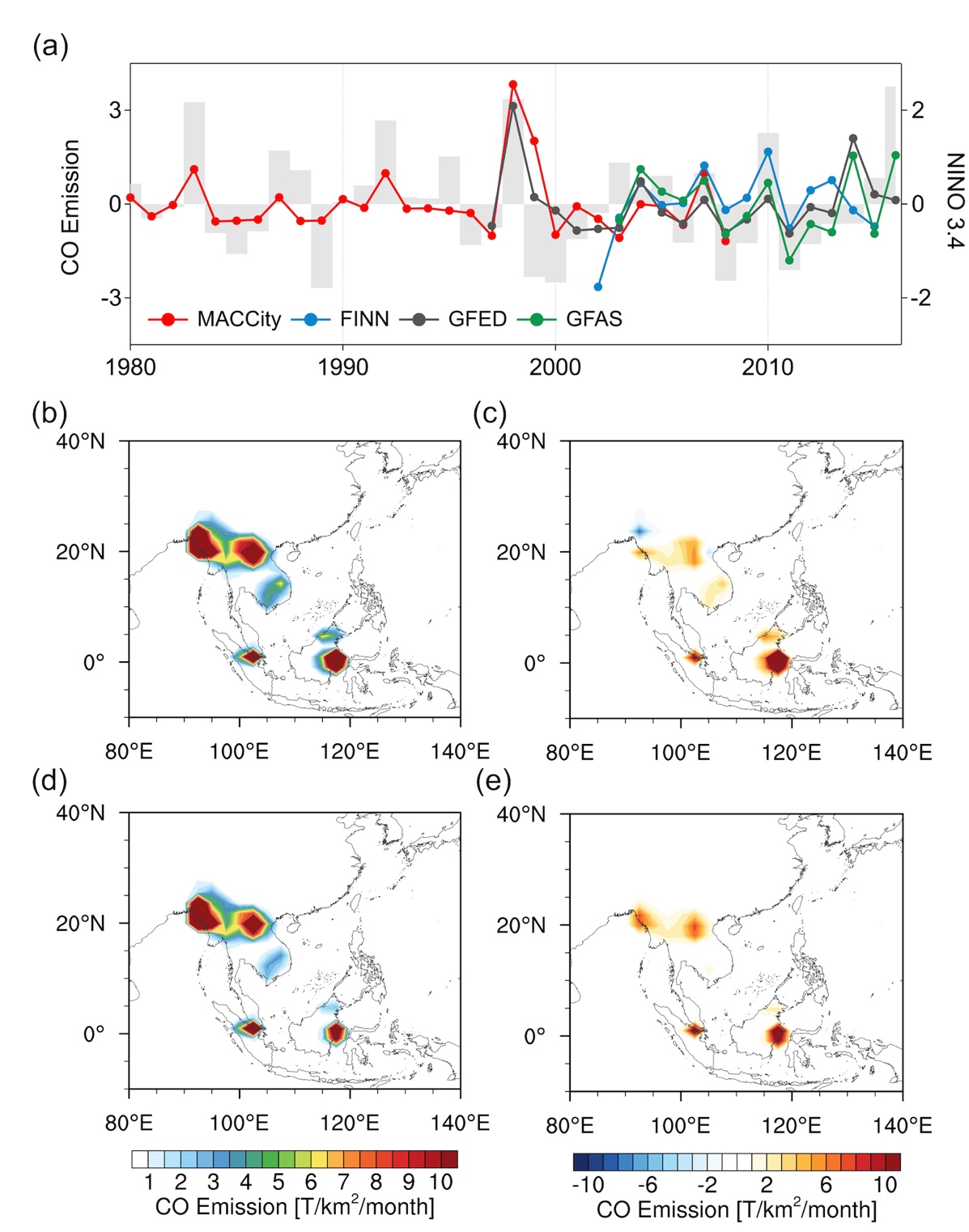


**Supplementary Figure 3.** Comparison of CO emissions from biomass burning in El Niño and La Niña springs. (a) Time series of normalized CO emissions in spring over Southeast Asia from different inventories. (b) CO emissions from MACCity in El Niño springs (March-April) during 1980-2008. (c) Difference of CO emissions between El Niño and La Niña springs. (d), (e) The same as (b), (c) but with the 1998/99 super El Niño/La Niña events excluded.


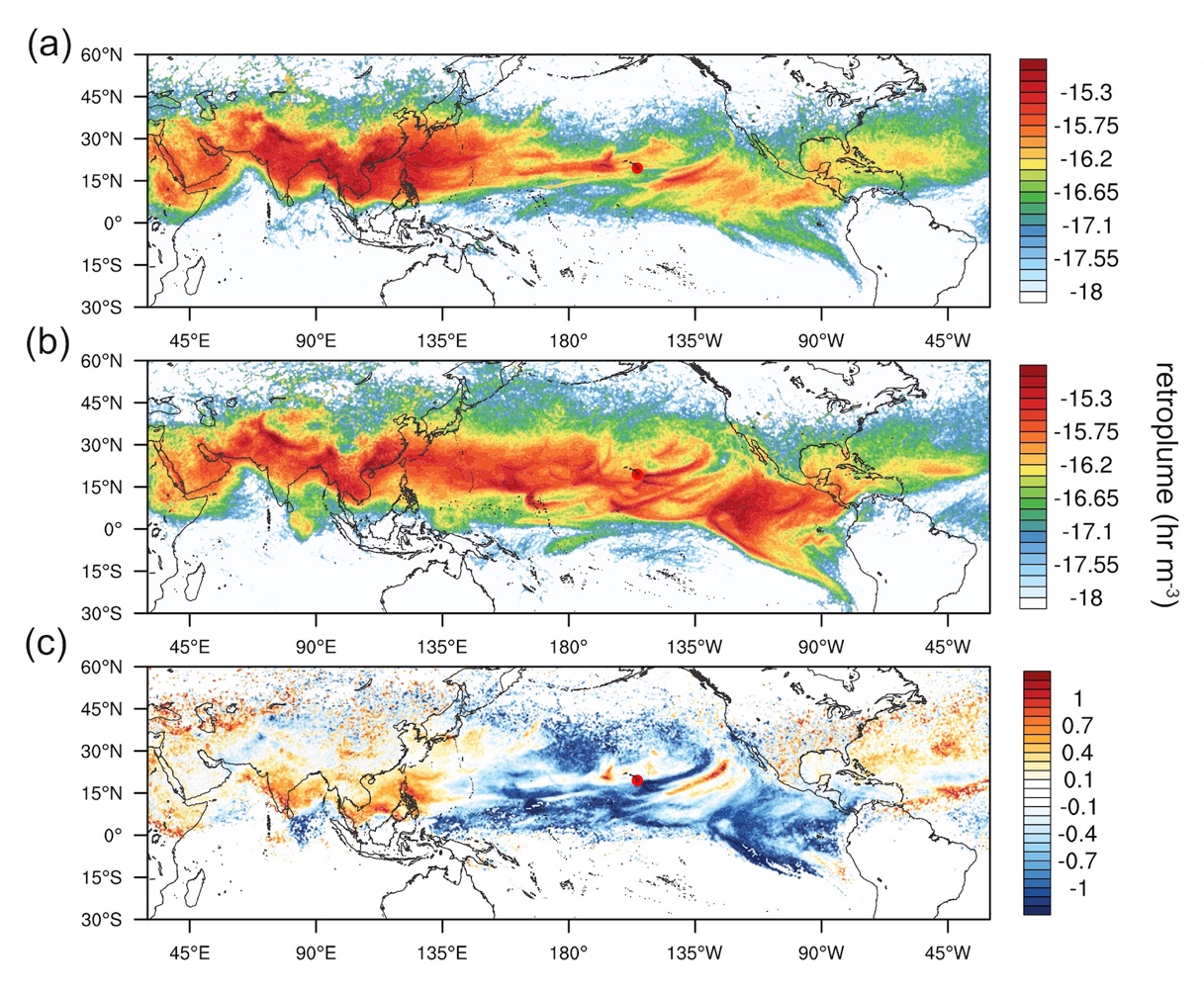


**Supplementary Figure 4.** 20-days backward Lagrangian retroplumes for MLO air masses in different ENSO phases. The spatial pattern of potential source regions for air masses measured at MLO in (a) El Niño springs, (b) La Niña springs, and (c) their difference. Red circles mark the location of MLO.


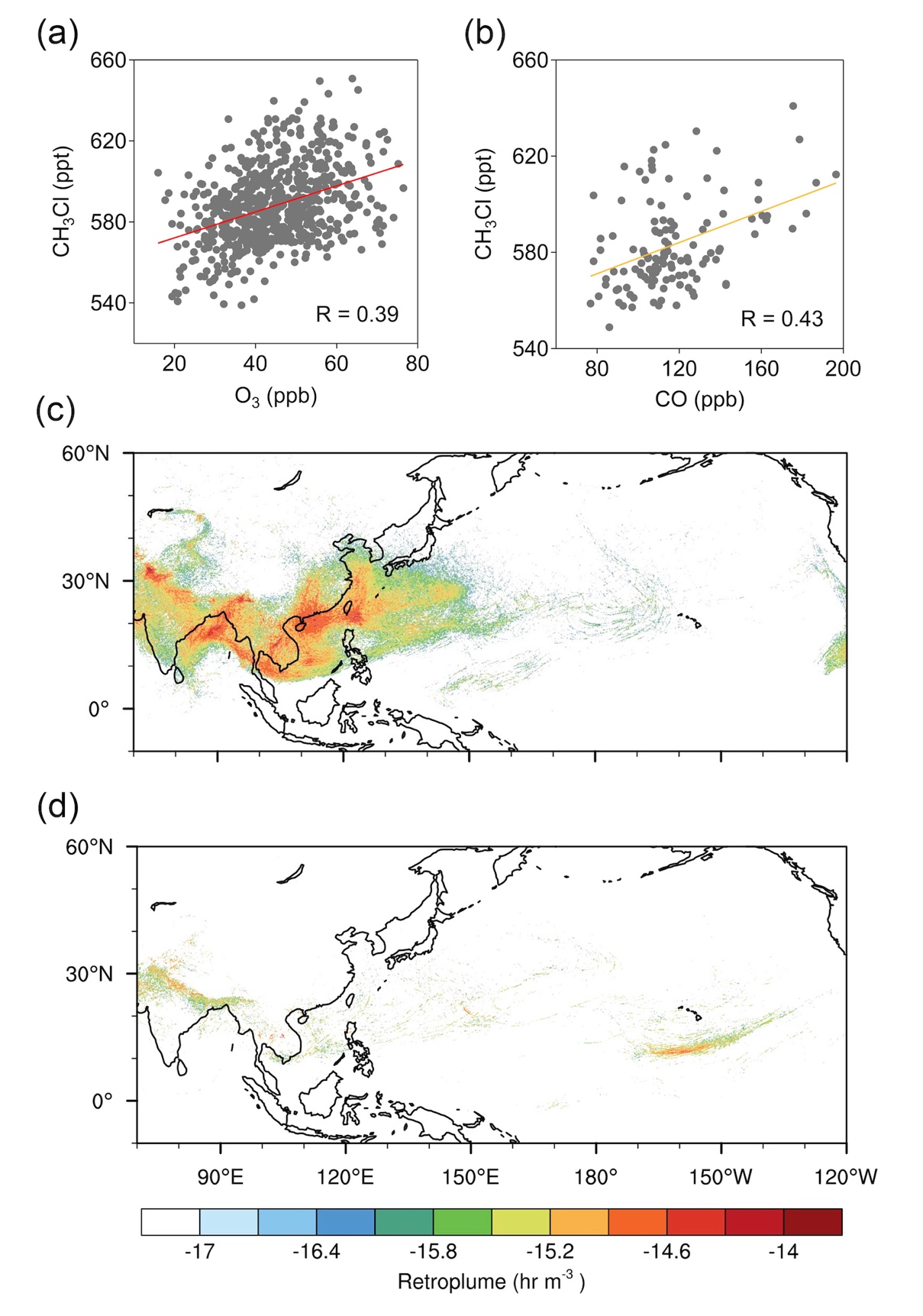


**Supplementary Figure 5.** Correlation of MLO trace gases and the potential source contribution in ENSO springs. (a) Scatter plot of nighttime mean CH_3_Cl and O_3_. (b) Same as (a) but for CO. The linear regression lines are also shown. (c) Potential source regions of air masses at MLO with CH_3_Cl higher than its upper quartile in El Niño springs. (d) Same as (c) but for La Niña springs.


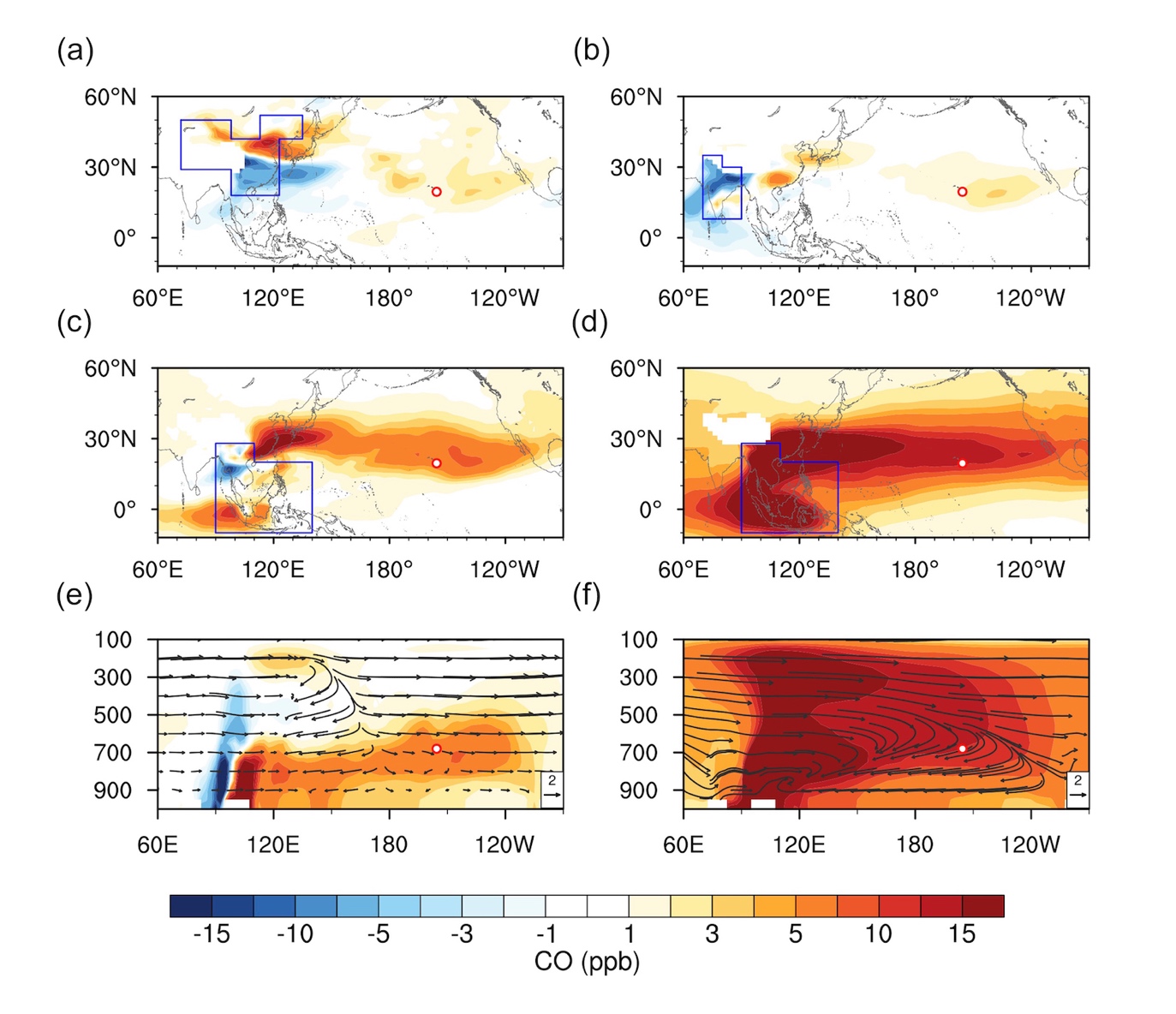


**Supplementary Figure 6.** The same as Figure 4 but for CO.


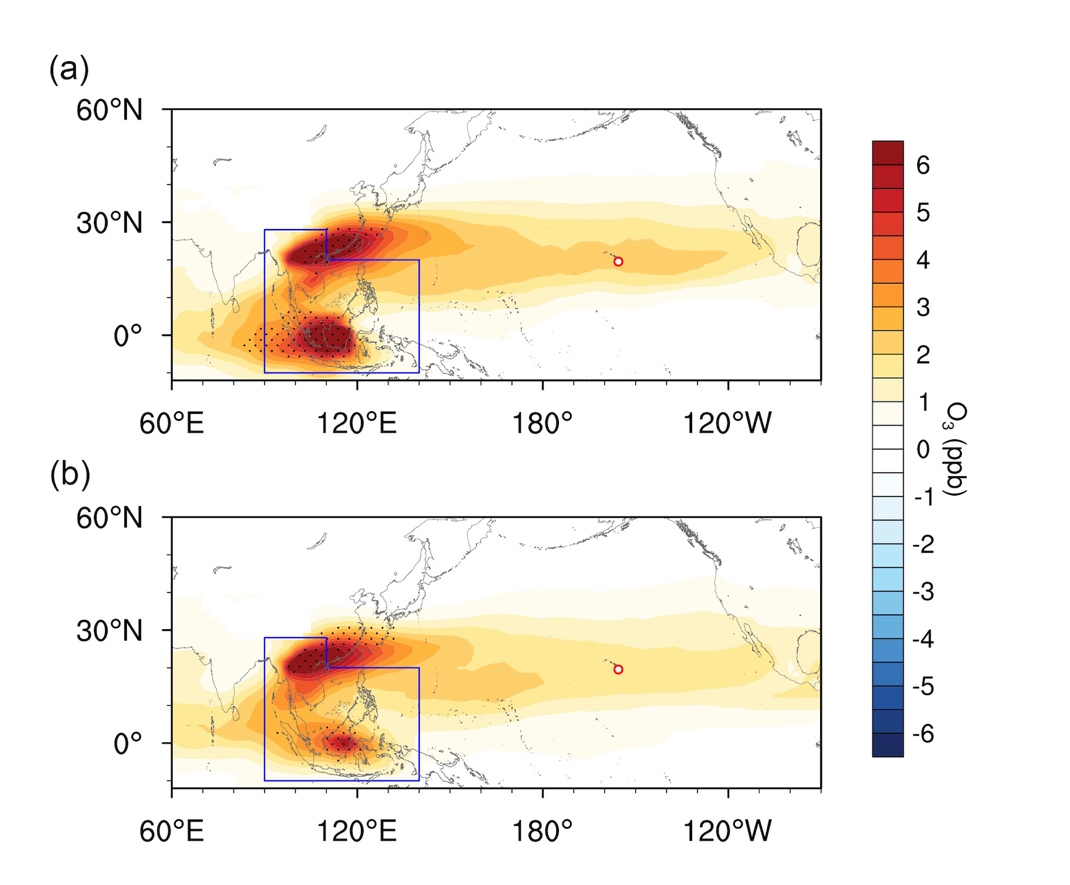


**Supplementary Figure 7.** Contributions of intensity variability of Southeast Asia biomass burning to O_3_ in ENSO conditions. (a) O_3_ response to SE BB in El Niño springs. The results were derived based on ElNino-ENSEBB and ElNino_LNSEBB runs in Supplementary Table 1. (b) The same with (a) but in La Niña springs. SE Asia is denoted by blue polygons. Red circles mark the location of MLO. The dotted areas indicate statistical significance with 90% confidence.


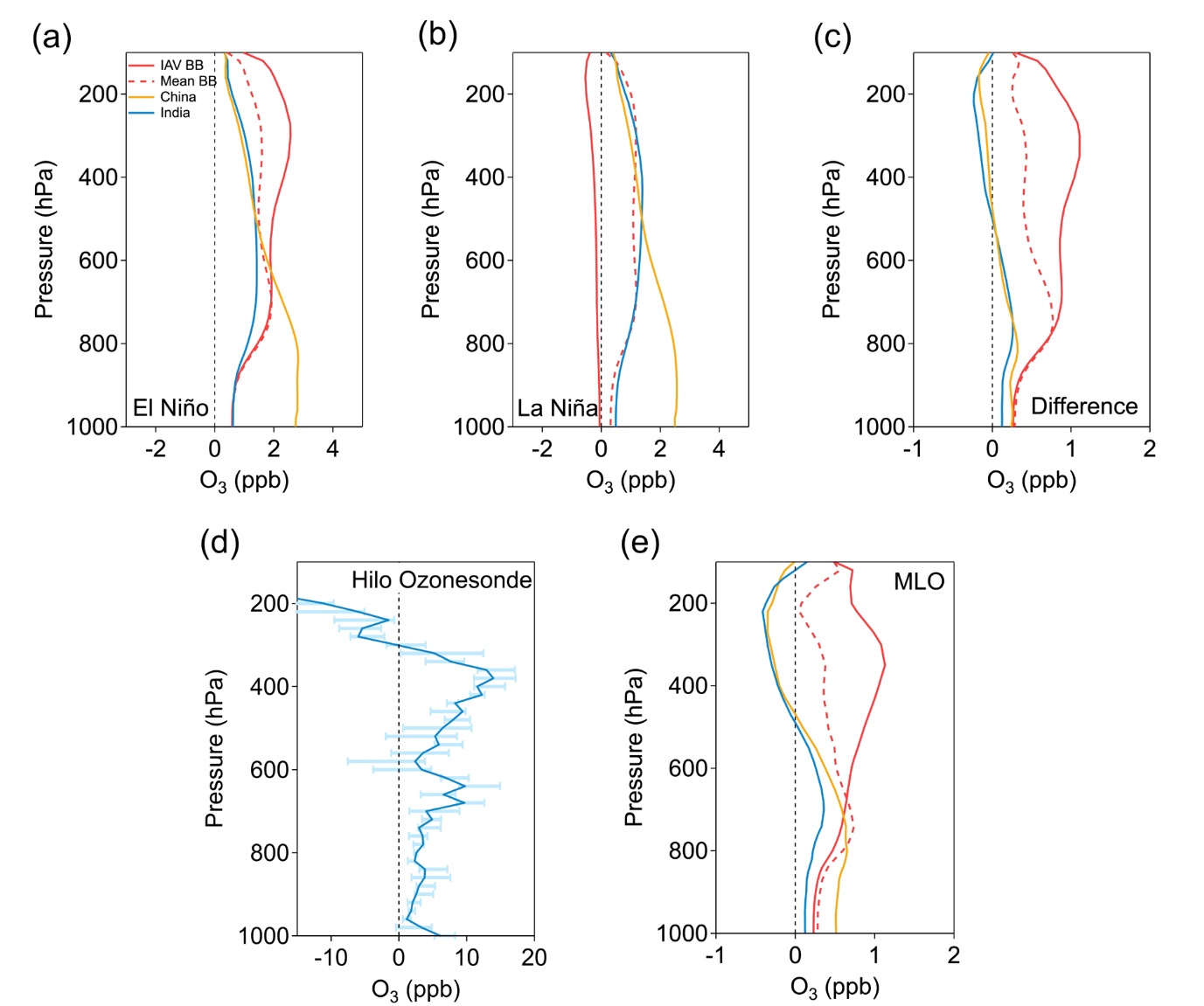


**Supplementary Figure 8.** Vertical profiles of ENSO-induced O_3_ anomalies from CESM simulations and ozonesonde measurement at MLO. (a)-(c) Source contributions to the ozone profile over the subtropical polluted band (averaged over 10 – 30 °N, 120 °E – 120 °W) in El Niño springs (a), La Niña springs (b) and their differences (c) in the MERRA driven CESM simulations. (d) ENSO composites of O_3_ profiles over Hilo, Hawaii. (e) Same as C but for that over MLO (15 – 20 °N, 155 – 160 °W) in CESM simulations.

**Supplementary Table 1**. Summary of model configurations and experiments design in CESM CAM-Chem simulations.

| **Exp. No.** | **Exp. Name** | **Meteorology** | **SST** | **Anthropogenic emissions** | **Biomass burning emissions** |
| --- | --- | --- | --- | --- | --- |
| 1 | ElNino_SST | Simulated online  (30 levels) | El Niño SST composites | Averaged in  2004-2008 | Averaged in  1980-2008 |
| 2 | LaNina_SST |  | La Niña SST composites |  |  |
| 3 | ElNino_BASE | MERRA reanalysis – 5 El Niño years  (56 levels) | Climatology | 2006 | 2006 |
| 4 | ElNino_ENSEBB |  |  | 2006 | El Niño mean emissions over SE Asia; 2006 elsewhere |
| 5 | ElNino_LNSEBB |  |  | 2006 | La Niña mean emissions over SE Asia; 2006 elsewhere |
| 6 | ElNino_SEclmBB |  |  | 2006 | Climatology emissions over SE Asia; 2006 elsewhere |
| 7 | ElNino_noSEBB |  |  | 2006 | No emissions over SE Asia; 2006 elsewhere |
| 8 | ElNino_ENICPBB |  |  | 2006 | El Niño mean emissions over Indochina Peninsular; 2006 elsewhere |
| 9 | ElNino_noICPBB |  |  | 2006 | No emissions over Indochina Peninsular; 2006 elsewhere |
| 10 | ElNino_noChina |  |  | No emission over China; 2006 elsewhere | 2006 |
| 11 | ElNino_noIndia |  |  | No emission over India; 2006 elsewhere | 2006 |
| 12 | LaNina_BASE | MERRA reanalysis – 5 La Niña years  (56 levels) | Climatology | 2006 | 2006 |
| 13 | LaNina_ENSEBB |  |  | 2006 | El Niño mean emissions over SE Asia; 2006 elsewhere |
| 14 | LaNina_LNSEBB |  |  | 2006 | La Niña mean emissions over SE Asia; 2006 elsewhere |
| 15 | LaNina_SEclmBB |  |  | 2006 | Climatology emissions over SE Asia; 2006 elsewhere |
| 16 | LaNina_noSEBB |  |  | 2006 | No emissions over SE Asia;  2006 elsewhere |
| 17 | LaNina_LNICPBB |  |  | 2006 | La Niña mean emissions over Indochina Peninsular; 2006 elsewhere |
| 18 | LaNina_noICPBB |  |  | 2006 | No emissions over Indochina Peninsular; 2006 elsewhere |
| 19 | LaNina_noChina |  |  | No emission over China; 2006 elsewhere | 2006 |
| 20 | LaNina_noIndia |  |  | No emission over India; 2006 elsewhere | 2006 |
| 21 | FSDSMAM_hist | MERRA reanalysis (1981-2008)  (56 levels) | Climatology | Historical | Historical |
| **Source** | | **Contribution Analysis** | | | |
| Anthro. emis. of China | | ( [ElNino_BASE] - [ElNino_noChina] ) – ( [LaNina_BASE] - [LaNina_noChina] ) | | | |
| Anthro. emis. of India | | ( [ElNino_BASE] - [ElNino_noIndia] ) – ( [LaNina_BASE] - [LaNina_noIndia] ) | | | |
| BB emis. of SE  (ENSO circulation) | | ( [ElNino_SEclmBB] - [ElNino_noSEBB] ) – ( [LaNina_SEclmBB] - [LaNina_noSEBB] ) | | | |
| BB emis. of SE  (intensity variability) | | { ( [ElNino_ENSEBB] - [ElNino_LNSEBB] ) + ( [LaNina_ENSEBB] - [LaNina_LNSEBB] ) } / 2 | | | |
| BB emis. of Indochina Peninsular | | ( [ElNino_ENICPBB] - [ElNino_noICPBB] ) – ( [LaNina_LNICPBB] - [LaNina_noICPBB] ) | | | |
| BB emis. of Indonesia | | ( [ElNino_ENSEBB] - [ElNino_ENICPBB] ) – ( [LaNina_LNSEBB] - [LaNina_LNICPBB] ) | | | |

| **Inventory** | **Period** | **# of ENSO Events**  **(El Niño/La Niña)** | **El Niño**  **(kg/km^2^/month)** | **La Niña**  **(kg/km^2^/month)** | **Difference** |
| --- | --- | --- | --- | --- | --- |
| MACCity | 1980-2008 | 9/8 | 5.3E+04 | 3.4E+04 | 57% |
| GFED4 | 1997-2016 | 6/5 | 2.9E+04 | 1.9E+04 | 52% |
| FINN | 2002-2015 | 4/3 | 7.7E+04 | 6.1E+04 | 26% |

**Supplementary Table 2.** Difference of CO emissions in El Niño/La Niña spring over Southeast Asia from different inventories.
